# Factors Associated With CT Scan Repetition in Pediatrics and Its Relationship With Cancer Risk: A Systematic Review and Meta-Analysis

**DOI:** 10.1177/15593258261419666

**Published:** 2026-04-16

**Authors:** Tahani Al-Shangeeti, Rozilawati Ahmad, Salah A. Alshehade, Maisa Elzaki, Fahad A. Alothaim, Walaa Alsharif, Khaled Soliman, Amjad Al-Shangeeti, Bandar Al-Shamrani, Abdullah Hanbali, Tariq Almuzaini, Mohammed Alnasser, Taher Althubyani, Abdullah Aljohani, Ahmed Mahdi Baba, Amal Zaki Alruwaili, Hamid Osman Hamid Osman, Mohammed Abdullah Alshawsh

**Affiliations:** 1 Faculty of Health Sciences, 61775 Universiti Kebangsaan Malaysia, Kuala Lumpur, Malaysia; 2 Department of Pharmacology, Faculty of Pharmacy, Universiti Sultan Zainal Abidin, Terengganu, Malaysia; 3 Department of Diagnostic Radiology, College of Applied Medical Sciences, 123305 Taibah University, Madinah, Saudi Arabia; 4 Saudi Food and Drug Authority Medical Device Sector, Radiological Health (SFDA), Riyadh, Saudi Arabia; 5 Medical Physics Department, 37853 Prince Sultan Military Medical City (PSMMC), Riyadh, Saudi Arabia; 6 Pediatric Department, King Faisal Specialized Hospital and Research Centre, Jeddah, Saudi Arabia; 7 Medical Physics Department, 37846 King Fahad Armed Forces Hospital, Jeddah, Saudi Arabia; 8 Pharmacy Department, 158232 Prince Sultan Armed Forces Hospital (PSAFHM), Madinah, Saudi Arabia; 9 Medical Physics Department, 158232 Prince Sultan Armed Forces Hospital (PSAFHM), Madinah, Saudi Arabia; 10 Radiology Department, 158232 Prince Sultan Armed Forces Hospital (PSAFHM), Madinah, Saudi Arabia; 11 Clinical and Molecular Microbiology Laboratories, 48132 King Abdulaziz University Hospital, Jeddah, Saudi Arabia; 12 Radiological Sciences Department, College of Applied Medical Sciences, 125895 Taif University, Taif, Saudi Arabia; 13 Department of Pharmacology, Faculty of Medicine, 37447 Universiti Malaya, Kuala Lumpur, Malaysia; 14 Department of Paediatrics, 2541 Monash University, Clayton, VIC, Australia

**Keywords:** computed tomography, CT scan, cancer risk, radiation exposure, pediatric radiation, cumulative doses, systematic review, meta-analysis

## Abstract

**Background:**

Rising use of pediatric CT scans has heightened concerns about radiation exposure compared to non-ionizing imaging modalities. This systematic review investigated factors contributing to repeat CT scans in children and assessed their association with cancer risk.

**Main body:**

Main body: A comprehensive search of Web of Science, Scopus, and PubMed identified 30 eligible studies, with five studies involving over seven million participants included in the meta-analysis. CT exposure was associated with a significantly increased overall cancer risk (RR = 1.49, 95% CI: 1.44-1.54). Risk of brain tumors was significantly elevated (RR = 1.55, 95% CI: 1.22-1.97), whereas evidence for leukemia was less conclusive (RR = 1.23, 95% CI: 0.72-2.12). A dose-response relationship was observed, with patients receiving two or more CT scans showing substantially higher cancer risk (RR = 2.51, 95% CI: 1.74-3.61) compared with a non-significant risk for those receiving only one scan (RR = 1.07, 95% CI: 0.73-1.56).

**Conclusion:**

These results highlight the need for practical pediatric CT guidelines. CT scans should be performed only when clinically justified, using optimized low-dose protocols and non-ionizing imaging alternatives when appropriate. Future research should develop evidence-based recommendations that balance diagnostic benefits with the long-term risks of radiation exposure, ensuring safe and effective imaging practice for children.

## Graphical abstract

**Graphical Abstract:**
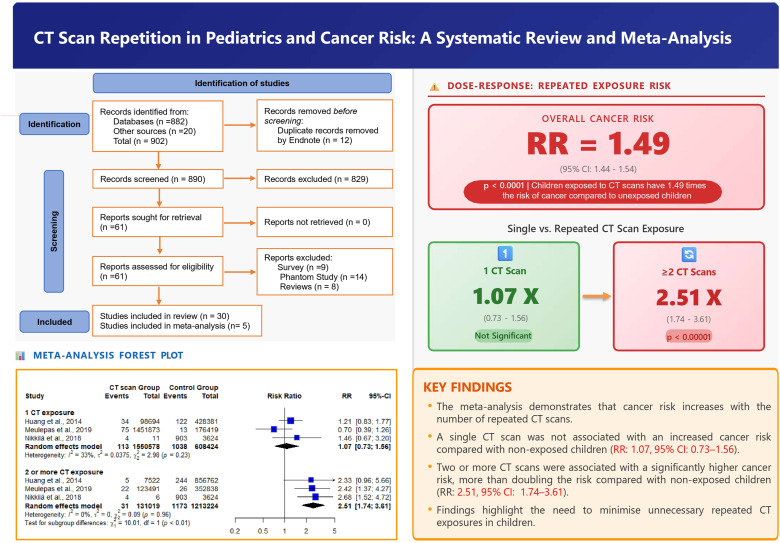

## Background

Computed tomography (CT) is an advanced medical imaging technique that uses X-rays and computer processing to obtain detailed cross-sectional images of the body.^1^ CT scanning provides important diagnostic information to assist medical evaluation and treatment planning. However, there are concerns about radiation exposure risks from CT scans, especially in more radiosensitive pediatric patients.^2^ Ionizing radiation from medical imaging can damage DNA and potentially cause cancer. Pediatric patients are especially vulnerable to radiation risks, given their developing bodies and longer life expectancy for potential radiation-induced cancer development.^3^ Over the last two decades, there has been a significant increase in the use of pediatric CT scans. CT scans emit ionizing radiation levels that are substantially higher than those of conventional radiography by a factor of 100-500.^4^ This heightened exposure to ionizing radiation has raised concerns regarding its potential correlation with increased cancer risk.^5^ Several studies have found increased cancer risks associated with CT scan radiation, particularly in pediatric patients. A cohort study found that pediatric patients who underwent multiple CT scans had a small but real increase in leukemia and brain cancer risks. Another retrospective cohort study indicated increased lymphoma, leukemia, lung, and liver cancer risks in pediatric patients receiving CT scans compared with those who did not.^6^ These risks highlight the need to limit unnecessary CT scans in pediatric patients. However, recent studies have indicated an increased utilization of CT scans in pediatric patients.^7^ Repeating CT scans further amplifies the cumulative radiation exposure and potential cancer risk.^3^

Despite concerns regarding pediatric CT radiation, the factors influencing the unnecessary repetition of CT scans in pediatric patients remain unclear. A few studies have examined the trends and predictors of repeat CT scans in adults.^8^; however, limited evidence exists for pediatric patients. Understanding the factors linked to unnecessary repetitive CT scanning in pediatric patients is crucial for optimizing its use and preventing excessive radiation exposure. Prior studies have examined the cancer risks associated with pediatric CT scans,^9^ but have not focused on the factors linked to repetitive scans. Understanding the predictors of repeat CT scans in pediatric patients is crucial for optimizing scan utilization and limiting unnecessary radiation exposure.

Current literature highlights significant gaps in understanding the factors contributing to CT scan repetition in pediatric settings, raising concerns due to the increasing use of CT in pediatric practice and its potential long-term health implications.

Therefore, this systematic review aimed to identify patient-related, provider-related, and health system-related factors linked to the repetition of CT scans in pediatric patients. In addition, the meta-analysis aimed to assess cancer risk in pediatric patients with repeated CT scans compared to those with single scans. Lastly, this study aimed to identify effective strategies for minimizing unnecessary repeat CT scans in pediatric cases.

## Methods

### Protocol and Registration

This systematic review followed the latest guidelines outlined in the Preferred Reporting Items for Systematic Reviews and Meta-Analyses Statement. We registered the review protocol in the International Prospective Register of Systematic Reviews (PROSPERO) database, which can be accessed at https://www.crd.york.ac.uk/PROSPERO, (ID: CRD42022342579). This protocol was published in 2023.^10^

### Search Strategy

Relevant studies were retrieved using standard database search terms. A search was performed using three online databases (PubMed, Scopus, and Web of Science) to identify pertinent studies. Google Scholar was employed to explore grey literature to guarantee the inclusion of all relevant studies. This systematic review included studies published in English between 2012 and 2024 exclusively. Two reviewers independently searched the relevant articles. Boolean AND/OR operators were used to connect the following search terms: Cumulative AND doses OR radiation AND dose) AND (CT-scan OR “CT scan” OR “CT scanning” OR “Computed Tomography”) AND (Tumor * OR cancer* OR Malignant* OR Tumor*) AND (Pediatric* OR Paediatric* OR child* OR infant*).

### Selection Process of Included Studies

The initial article screening process was conducted separately by two reviewers. They independently assessed the titles and abstracts of the studies and categorized them into three groups: relevant, irrelevant, and uncertain. This systematic review and meta-analysis included studies that focused on pediatric patients aged <18 years who were exposed to CT scan radiation. It included any type of observational study, such as cohort studies, case-control studies, or experiments, if the specified population and outcomes were examined. The inclusion criteria specifically covered studies that investigated the relative risk of cancer incidence associated with single or repeated CT scan exposure among pediatric patients; the prevalent cancer types linked to these exposures; and the rates, cumulative doses, and reasons for multiple CT scan exposures. Studies involving hybrid CT modalities, such as PET/CT and SPECT-CT, phantom studies, and systematic reviews or meta-analyses, were excluded from the analysis. Peers deemed irrelevant by both reviewers were excluded. Following this, four additional reviewers examined the full text of the remaining studies using the eligibility criteria, and only studies that met all criteria were included. In the event of disagreement, the two reviewers held discussions to reach a consensus before making their final decisions. If any confusion or disagreement persisted, a third opinion was sought from the other reviewers and a final decision was made upon reaching consensus.

### Data Extraction and Risk of Bias Assessment

To improve the clarity and quality of the language, a standardized data extraction form in the form of a Google Spreadsheet was employed to enhance the consistency and accuracy of data recording. The following information was extracted and documented: Study ID, author name, publication type, journal name, Title, Year, Country, number, age, and gender of children in the exposure group; number, age, and gender of children in the non-exposure group (comparator); CT scan details, including the number of exposures, CT scan repetition rate, duration, cumulative dose, organs/tissues scanned, the reason for scanning, frequency of scanning, and duration between multiple CT scan radiations; cancer incidence rate, expressed as risk ratio, odds ratio (OR), or relative risk; cancer types; the relationship between cancer risk and multiple CT scans; study duration; and risk factors.

Potential bias was assessed using the Newcastle-Ottawa Scale (NOS) for observational studies, a tool designed to evaluate the quality of non-randomized studies within a systematic review context.^11^ NOS offers a thorough evaluation of the strength of evidence about the possible association between exposure and outcome. It assessed two crucial aspects: (i) the selection of appropriate studies to address the exposure-outcome relationship in question and (ii) how well these studies estimated the exposure-outcome relationship without significant bias risks. Each study’s evaluation was based on the available data. The NOS assessment concentrates on various key domains, including the selection of study groups, comparability of these groups, and ascertainment of outcomes. Specific criteria were used for each domain to gauge the risk of bias. Two independent assessors conducted the NOS-based risk assessment, and any disparities or disagreements were addressed through discussion involving a third assessor if necessary. This meticulous process guarantees the reliability and validity of the NOS assessment. The study groups were thoroughly scrutinized to ensure that they accurately represented the target population and were matched suitably in terms of pertinent characteristics. The comparability of the groups was evaluated to verify the similarity of all aspects between the groups, except for the exposure being investigated, which helped minimize the potential confounding factors. Furthermore, the outcome ascertainment was reviewed to confirm unbiased and appropriate outcome measurements and assessments. Specific criteria for each domain considered factors such as sample size, study design, potential sources of bias, and statistical methodologies employed. This systematic review was assessed using the Preferred Reporting Items for Systematic Reviews and Meta-analyses (PRISMA) checklist, which ensures the inclusion of high-quality studies and adherence to rigorous reporting standards.^12^

### Strategy for Data Synthesis

The approach to synthesizing data involved first gathering both quantitative and qualitative data from each study. The qualitative findings are described in the text and tables. However, for quantitative data related to the outcomes, a meta-analysis was performed by combining homogeneous data across studies. The effect size was calculated as the risk ratio, based on the method reported in the included studies. The choice between random- and fixed-effects models was determined by primary studies to account for variations when calculating the overall estimate.

To assess heterogeneity, we used the I^2^ measure, which can be evaluated visually using a forest plot. An I^2^ value between 0 and 30% indicated low heterogeneity, 30-65% suggested moderate heterogeneity, and >65% indicated substantial heterogeneity.^13^ We evaluated publication and selection biases by constructing a funnel plot and conducted a sensitivity analysis to test the strength and reliability of our findings.

## Results

### General Characteristics of the Included Studies

**Figure 1.**
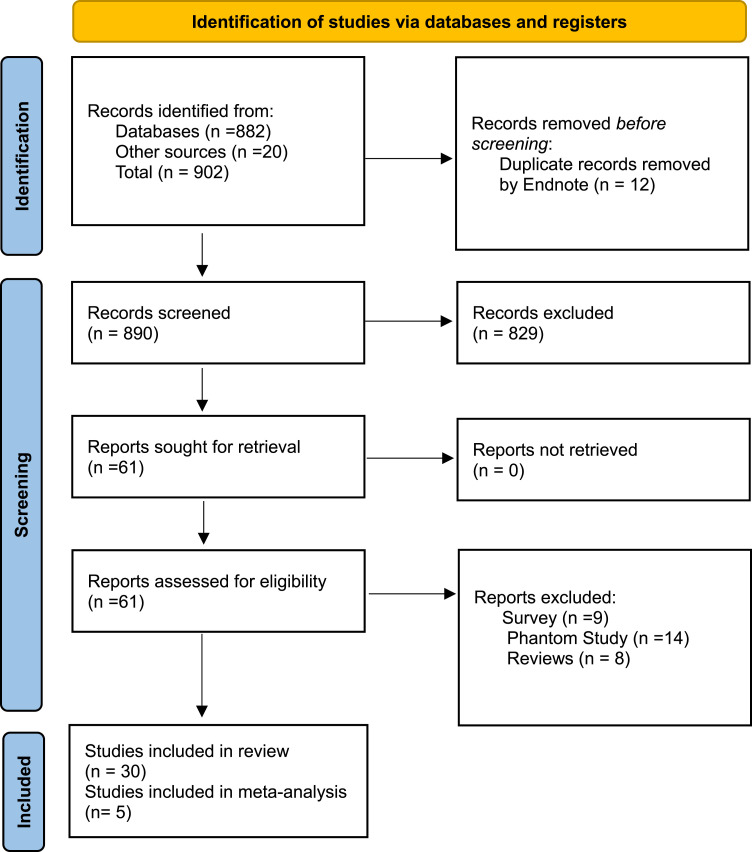
The PRISMA flow chart summarizes the systematic review process adapted from Ref. 12

A total of 902 records were initially identified from databases and other sources. After removing 12 duplicate records, 890 records were screened, leading to the exclusion of 829 records based on the predefined criteria. Subsequently, 61 successful reports were retrieved. Following retrieval, 61 full texts were assessed for eligibility, resulting in the exclusion of 31 reports, including nine surveys, 14 phantom studies, and eight reviews. Ultimately, 30 studies were included in the review, of which 5 were included in the meta-analysis (Figure 1).

These studies span a decade, from 2012 to 2023, underscoring the contemporary significance of our research topic. Approximately half of these studies (n = 17) were published during the most recent period (2018-2023), highlighting the dynamic evolution of this research domain (Table 1). Upon examining the regional distribution of publications in this systematic review, we identified discernible disparities. Asia emerged as the most prolific contributor, accounting for 38% of publications. By contrast, Africa exhibited the lowest representation, contributing only 3% of the publications, underscoring a marked regional discrepancy in research output.

**Table 1.** Characteristics of the Included Studies

No.	Ref	Country	Study design	Participants			CT protocols	Type of cancers
				Number	Age (year)	Male/female		
1.	14	China	Retrospective study	922 children	≤15	626/296	Head, paranasal sinus, chest	Thyroid
2.	7	USA (diverse racial/ethnic)	Retrospective observational	4,857,736 = observation 744 = for dose calculation	<15	Girls = 50%	Head, chest, abdomen/pelvis, and spine	Solid cancer and leukemia
3.	15	Netherland	Cohort retrospective	168,394	≤18		Head, chest, abdomen/pelvis, and extremities	Brain leukemia
4.	1	Taiwan	Retrospective	2756	≤16	1439/1094	N/A	Leukaemia, intracranial malignancy, lymphoma
5.	16	Japan	Retrospective	763	0-19	424/339	Brain/head and neck, thorax, and abdomen	N/A
6.	17	Canada	Prospective multicenter cohort study	4060	0-16	2618/1442	Brain	Trauma
7.	18	UK	Cohort	1073	0-19		Head, chest, abdomen, and pelvis, non-head scan	
8.	6	Taiwan	Cohort	24,418	<18		Head	Brain tumors, leukemia
9.	18	Japan	Retrospective	980	<15	544/436	Head	
10.	5	Australia	Cohort	10,939,680	0-19	357119/323092	Brain, facial bone, chest, extremities, abdomen, pelvis, spine, neck, other	Solid cancer, leukemia
11.	19	Sudan	Retrospective	182	1.12 month - 10 years		Head, chest, abdomen	
12.	20	Finland	Cohort	4372	<15	2272/2100	Head CT	Leukemia
13.	21	Brazil	Cohort	1006	0 - 17.83	545/461	Head	Brain tumor
14.	22	France	Cohort	27,362	<5	15869/11492	Head, neck, chest, abdomen, pelvis, extremities	Risk of cancers
15.	23	Oman	Retrospective	142	4-87	52/90	Head, sinuses	
16.	24	Finland	Retrospective	48,989	0-15	2766/21329	Head, neck, chest, abdomen, pelvis, extremities	N/A
17.	9	England	Retrospective cohort	78,604 leukemia & 17,6587 brain tumors	0-15	Leukemia: 953,634/764,937 Unknown = 2413 Brain: 657,169/529,372 Unknown = 1666	Head, abdomen, pelvis, chest	Leukemia, brain
18.	25	Britain	Retrospective cohort	180,000	<22		Head CT scan	Leukemia, brain tumors
19.	26	Japan	Retrospective	138,532	0-10	86,305/52,226	Head	Brain, CNS
20.	3	France	Cohort	67,274	0-10	N/A	138,532 head CT examinations	Centre nervous system tumor, lymphoma, leukemia
21.	27	Spain	Cohort retrospective	9.7 million	0-20	52,283/42,324	Head, face, cervical spine,	Brain
22.	28	Canada	Retrospective	157	<18	115/42	Head, face, spine, body (chest, abdomen, pelvis), extremities	N/A
23.	29	UK	Cohort	1073	0-19	N/A	1. Head 2. Chest 3. Abdomen and pelvis	N/A
24.	30	Australia	Cohort	12,194,798	0-85	5,618,995/6,575,803	Abdomen, pelvis, chest, extremity, facial, bones, head, neck, spine spiral angiography	N/A
25.	31	USA	Retrospective analysis	487	5-13	301/186	Head CT, cervical spine, abdomen, chest	N/A
26.	32	Iran	Retrospective	932	18-57	544/388	Abdomen pelvis, routine chest, chest HR CT, brain, sinise examination	N/A
27.	33	Turkey	Cohort study	561	<18	373/188	Head CT	Leukemia, CNS
28.	34	Switzerland	Cohort study	522	0-15	291/231	Chest CT scan protocol	Leukemia, colon liver, lung, ovary, stomach, breast, uterus
29.	35	Iran	Cross-sectional study	90	<20	N/A	Brain CT	Thyroid
30.	36	Iran	Retrospectives	19,800	0-15	2341/1610	Brain	Leukemia, brain tumor

**Figure 2.**
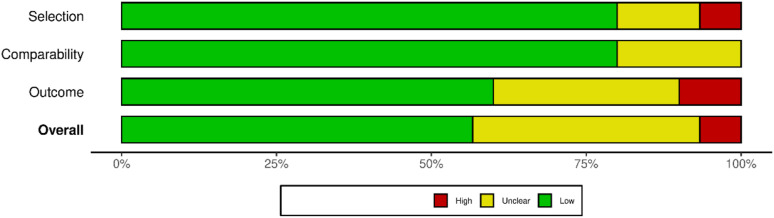
The overall risk of bias assessment

Furthermore, the overall risk of bias assessment showed that most of the studies had a low risk of bias (Figure 2). This was indicated by 16 studies that received scores of 7 or more. 12 studies scored between 5 and 6, indicating a moderate risk of bias, whereas two studies scored below 5, indicating a higher risk of bias (Supplemental Figure S1). Notably, all studies included in the meta-analysis had an overall low risk of bias.

### Risk of Cancer Associated with CT Scan in Pediatric Patients

**Figure 3.**
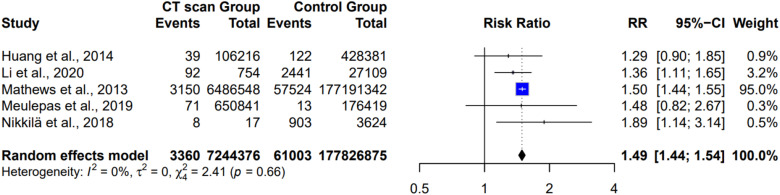
A meta-analysis examined the association between exposure to CT scans and cancer in pediatric patients

In this study, the association between CT scan exposure and cancer risk was examined by meta-analysis (Figure 3), which revealed that five studies were included,^1,5,6,15,20^ with a total of 7,244,376 participants in the CT-exposed group and 1,778,268,875 participants in the non-exposed group. There were 3360 cancer events in the CT-exposed group and 61,003 in the non-CT-exposed group. Using a random effects model, the meta-analysis found a pooled risk ratio (RR) of 1.49 (95% CI: 1.44 to 1.54, *P* < 0.00001), indicating a statistically significant increased risk of cancer among those exposed to CT scans compared to those not exposed. The study by Mathews et al contributed the largest weight to the analysis (95.1%)^5^; however, removing this study data from the meta-analysis does not affect the significant level and the RR was 1.39 (95% CI: 91.19 – 163, *P* < 0.00001). The risk ratios from the individual studies ranged from 1.29 to 1.89, suggesting an increased risk of cancer with CT scan exposure. Although not all were statistically significant, they consistently showed elevated cancer risk following CT scan exposure. The heterogeneity between the studies was low (I^2^ = 0%), indicating consistency of the results.

### Risk of Cancer Associated with CT-Scan Repetition and Cumulative Dose

**Figure 4.**
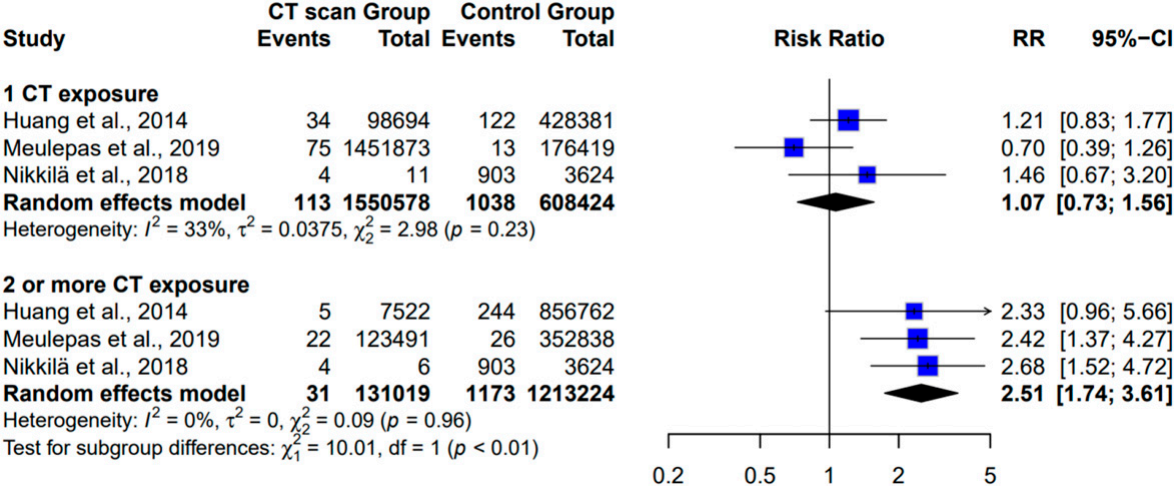
Subgroup analysis revealed that the cancer risk varied according to the number of repeat CT scans

Figure 4 shows the subgroup analysis of cancer risk according to the number of repeat CT scans. In the single repeat CT scan group, three studies with over 1.5 million participants found a non-significant pooled risk ratio of 1.07 (95% CI: 0.73 to 1.56, *P* = 0.75). Conversely, among children receiving two or more repeat CT scans, three studies with 7524 participants found a significantly increased risk ratio (RR = 2.51, 95% CI: 1.74 to 3.61, *P* < 0.0001), indicating that two or more CT scans were associated with a higher risk of cancer than one CT scan. Moderate heterogeneity was observed among studies with a single repeat CT scan (I^2^ = 33%), whereas low heterogeneity was observed among studies with two or more repeat CT scans (I^2^ = 0%). This finding provides evidence that cancer risk increases with the number of repeated CT scans in children.^5,6,20^

### Risk for Specific Types of Cancer

**Figure 5.**
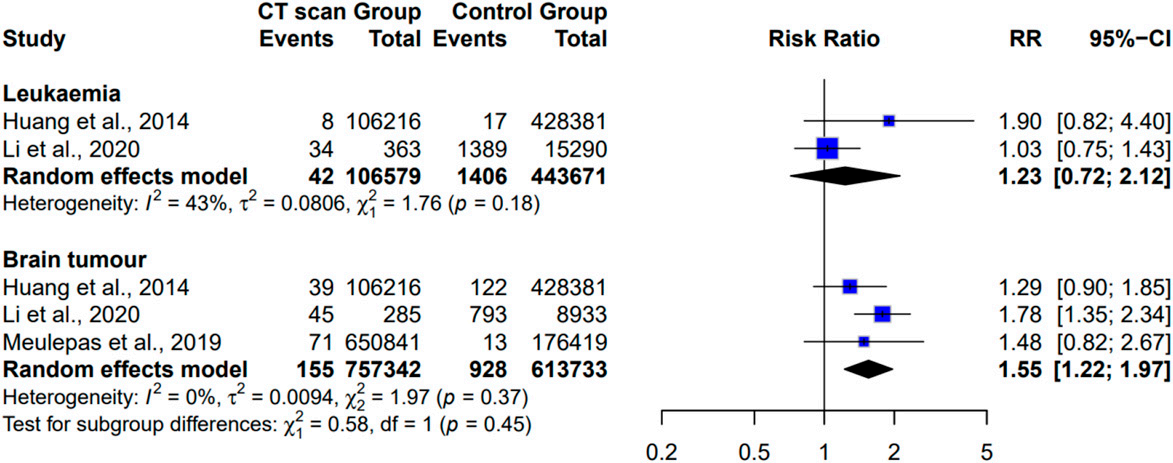
Subgroup analysis of cancer risk from CT scans varied according to cancer type

Figure 5 shows the subgroup analysis examining whether the cancer risk from CT scans differed by cancer type, including leukemia and brain tumors. For leukemia, two studies with 106,579 participants found a non-significant pooled risk ratio of 1.23 (95% CI: 0.72 to 2.12, *P* = 0.45), with moderate heterogeneity (I^2^ = 43%). In contrast, for brain tumors, three studies with 757,342 participants found a significantly increased pooled risk ratio of 1.55 (95% CI: 1.22 to 1.97, *P* < 0.00001), with low heterogeneity (I^2^ = 0%).

#### Leukemia

One study found that undergoing two to three repeat CT scans substantially increased the risk of blood cancer in children under five years of age, particularly with head CT scans. Furthermore, the risk of solid cancers is higher among younger patients and girls than among older children and boys.^7^ Regarding the risk of leukemia, the estimated lifetime attributable risk (LAR) was highest for head scans in children under 10 years of age and decreased as the pediatric population grew older. Specifically, for pediatrics under 5 years of age, the projected risk was 1.9 cases per 10,000 scans. However, for children aged 10 to 14 years, this risk dropped to 0.5 cases per 10,000 scans. In the case of abdomen/pelvis scans, the leukemia risk ranged from 0.8 to 1.0 cases per 10,000 scans, while for chest and spine scans, it was estimated to be between 0.4 to 0.7 cases per 10,000 scans. Interestingly, among pediatrics aged 10 to 14 years, abdomen/pelvis scans exhibited the highest associated risk of leukemia, at 1.0 cases per 10,000 scans. To contextualize this, it was estimated that one case of leukemia could potentially result from 5250 head scans performed in children under 5 years of age and from every 21,160 scans in pediatric patients aged 10-14 years.^7^ The anticipated number of future cancer cases, particularly in the context of leukemia and brain cancer, is a critical area of research and public health concern. Based on data from 19,800 brain CT scans conducted in Yazd Province, the projected number of future cases of brain cancer and leukemia were 4 and 1.7, respectively. These future estimates for the prevalence of leukemia and brain cancer were 1.8 and 0.75 for men, and 1.3 and 0.6 for women, respectively. Approximately one cancer case was attributed to childhood exposure that later developed into cancer, comprising 0.23 leukemia cases and 0.53 brain cancer cases. Examining the projected cancer rates within three distinct age groups, the total number of cases of brain cancer and leukemia was approximately 4.6 and 0.7, respectively.^37^ Furthermore, a corroborating study identified a positive correlation between leukemia brain tumors, and radiation exposure resulting from CT scans. The ERR per mGy was 0.036 (95% CI: 0.005-0.120) for leukemia and 0.023 (95% CI: 0.010-0.049) for brain tumors. This study revealed that the leukemia risk may nearly triple with cumulative doses of approximately 50 mGy delivered during CT scans in pediatric patients, whereas the risk of brain cancer may almost triple with cumulative doses of approximately 60 mGy.^9^ Conversely, another study found no association between the radiation dose to the bone marrow and the risk of all types of leukemia combined, with 44 cases analyzed. The ERR per 100 mGy was 0.21 (95% CI: −0.12 to 2.40) with a *P*-value of 0.68. This trend remained consistent for all leukemia and myelodysplastic syndromes (MDS) combined, with an ERR per 100 mGy of 0.04 (95% CI: −0.12 to 1.61) and a *P*-value of 0.92.^15^ In the case of the most prevalent subtype, precursor B-cell acute lymphoblastic leukemia, there was an evident heightened risk linked to radiation exposure, with an OR of 0.14 mGy. Moreover, the excess OR for individuals who underwent any CT scan vs those who did not was 2.25 for acute lymphoblastic leukemia and 2.88 for precursor B-cell, respectively. These findings underscore the notably higher risk associated with specific leukemia types among individuals exposed to CT scans.^5^

#### Solid Cancer

The estimated LAR of solid cancers decreases with increasing age, particularly for head and spinal scans. In contrast, the relationship between the abdomen/pelvis and chest scans was less consistent. Notably, the risk of solid cancers was found to be greater for females than for males, with the highest projected cases per 10,000 CT scans occurring for abdomen/pelvis scans (ranging from 25.8 to 33.9 cases for girls compared to 13.1 to 14.8 cases per 10,000 CT scans for males).^7^ When specifically assessing the cancer risks associated with various organs following medical imaging, an increase was observed in comparison with naturally occurring lifetime estimates. In female neonates, this increase was 1.8%, 0.45 %, 0.45 %, and 0.39 % in the thyroid, stomach, lungs, and breasts, respectively. When these organ-specific risk increments for the analyzed cancer sites were summed, a cumulative increase of up to 3.6% in female neonates and 2.1% in male neonates was observed compared with natural risks. However, for females aged 10 years, these organ-specific increases were less pronounced, with increments of 0.57% in the thyroid, 0.19% in the stomach, 0.24 % in the lungs, and 0.18% in the breasts, resulting in a 1.3% (0.7% in 10-year-olds) cumulative increase in cancer risk.^34^

##### Brain Tumors

A Taiwanese research study revealed that the hazard ratio (HR) for all types of brain tumors was significantly elevated, with a value of 2.56 (95% CI: 1.44-4.54, *P* < 0.01), in the cohort exposed to specific factors compared to the cohort without exposure. However, it is important to note that the overall risk of both malignant and benign brain tumors did not differ significantly between the two cohorts. Notably, the frequency of CT scans displayed a robust correlation with the incidence of all brain tumors, with the HR increasing from 2.32 to 10.4 (*P* < 0.001) compared to the unexposed cohort.^1^ Another study found that the risk of developing benign brain tumors in Taiwan was 2.97 times higher among patients who underwent head CT scanning procedures during childhood than among those who did not undergo CT scans during childhood. Additionally, participants aged 7-12 years are more susceptible to benign brain tumors, and overall, those aged 0-6 years have the highest risk.^6^ For all brain tumors, as well as malignant and non-malignant brain tumors considered separately, the relative risks notably increased, reaching two to four times the baseline risk in the highest dose category (120 mGy). The ERR per 100 mGy was 0.86 (95% CI: 0.20 to 2.22) for all brain tumors, 1.02 (95% CI: 0.01 to 4.30) for malignant brain tumors, and 0.78 (95% CI: 0.07 to 2.58) for non-malignant brain tumors. Importantly, there was no evidence of nonlinearity in these trends, as indicated by *P*-values of 0.72, 0.45, and 0.92 for the respective analyses.^15^ Cerebral tumors are the most common radiation-related cancers, accounting for 25% of future cancer cases.^29^ CT scan exposure increases the risk of various cancers, with higher risks being associated with specific solid, lymphoid, and hematopoietic cancers. The cumulative absolute excess incidence rate for all cancers was 9.38 per 100,000 person-years.^5^

##### Thyroid

The LAR of thyroid cancer incidence in a 5-year-old child who underwent a single-head CT scan was found to be 8.8 per 100,000 in girls and 1.3 per 100,000 in boys. In the case of chest CT scans, the LAR was notably higher at 22.4 per 100,000 for girls and 3.2 per 100,000 for boys. However, for patients aged 5-10 years who underwent chest CT, LAR ranged from 16.0 per 100,000 to 44.7 per 100,000. This wide range can be attributed to variations in the age of the exposed patients and the frequency of repeated CT scans.^14^ Additionally, a study reported that pediatric patients under the age of five years had an increased risk of thyroid cancer. According to the results of calculating the lifetime risk of thyroid cancer, the spiral technique has a much higher risk than the other techniques.^35^ Organ-specific cancer risks were found to increase by up to 1.8 % in the thyroid, 0.45 % in the stomach, 0.45 % in the lungs, and 0.39% in the breasts of female neonates.

### Factors Associated With CT-Scan Repetition in Pediatric Patients

Although it is essential to understand the key factors leading to the repetition of CT scans, there is a notable lack of comprehensive studies in the existing literature that address these factors. The factors identified in the available literature regarding the repetition of CT scans can be categorized into three main groups: clinical and technical (involving factors such as radiographer practices and scanning protocols) and patient-related factors such as motion during scanning (Table 2). A noteworthy cause of repeated CT examinations in pediatric patients is the diversity of imaging protocols, even within the same medical department. The inconsistent use of protocols by technologists can compromise image quality and diagnostic accuracy, necessitating additional scans. Furthermore, the application of adult protocols to pediatric patients, which do not account for their unique anatomical and physiological characteristics, often results in suboptimal imaging outcomes and requires repeated examinations. Therefore, it is imperative to establish standardized protocols tailored to pediatric patients to ensure optimal diagnostic results, while minimizing radiation exposure. Clinical factors also play a pivotal role in the repetition of CT examinations in the pediatric population. In resuscitation scenarios, multiple scans are often necessary to assess the effectiveness of resuscitative efforts and monitor changes in the patient’s condition over time. Repeat examinations are essential for re-evaluating injuries, particularly when initial imaging provides limited or inconclusive results. Additionally, changes in clinical status, such as worsening of symptoms or the emergence of new complications, may prompt the need for repeat CT examinations to facilitate accurate diagnosis and guide treatment planning. This systematic review also highlighted previous research indicating that patients with low Glasgow Coma Scale (GCS) scores may experience ongoing injury progression. Consequently, repeated imaging is recommended in such cases to monitor the evolving pathology and to adjust the treatment approach accordingly.

**Table 2.** Factors Contributing to Radiation Exposure Variability in Pediatric CT Scanning

Factors	Note
Inconsistent technologist practices	Some technologists apply identical CT parameters for both adults and pediatric, leading to elevated radiation doses in pediatric patients
Deficient technologist training	A significant issue arises from the inadequate training of technologists in pediatric dose optimization
Shielding absence	Radiosensitive organ shielding, such as thyroid, gonad, or eye lens shields, are not employed by hospitals during pediatric CT scans, leaving these organs susceptible to scatter radiation
Wide variability in previous studies	Past research has uncovered substantial variations in screening times, radiographic images, dose-length products, and effective doses in pediatric CT scans, indicating unnecessary radiation exposure
Variations across body regions	Minor dose variations were observed for brain and chest CT scans, but significant discrepancies were found for abdominal CT scans, likely stemming from differences in clinical indications
Insufficient knowledge among healthcare professionals	Variability in practice is exacerbated when technologists, radiologists, and referring physicians lack adequate knowledge about CT machine capabilities and radiation risks
Consistency in dose across CT modalities	The study found consistent dose values across different CT modalities in all three hospitals
Lack of optimization implementation	Examined hospitals did not implement CT dose optimization strategies, resulting in unnecessarily high radiation doses
Impact of CT scanner types	The choice of CT scanner type affects radiation dose, with dual-slice or four-slice CT scanners delivering lower doses but potentially compromising image quality, while higher-slice CT modalities provide doses that exceed what is necessary
Variability due to data and protocols	Variations in data provided in request forms and department protocols contribute to dose discrepancies
Significance of pediatric protocols	The use of adult techniques and protocols in some cases highlights the importance of dedicated pediatric protocols for CT examinations in pediatrics
Need for referral criteria and staff training	The study underscores the importance of establishing clear referral criteria and providing ongoing staff training in radiation protection concepts

Furthermore, it was reported that many patients undergo repeat CT scans primarily for injury reassessment rather than due to a change in their clinical condition.^28^ Head injuries stand out as a prominent reason for the repetition of CT scans in pediatric cases, ensuring the accuracy of the diagnosis^33^ revealed a significant disparity in radiation exposure levels. Pediatric head CT scans administered without following the specific pediatric protocol were associated with an average effective radiation dose that was twice as high as when the protocol was applied, measuring at 3.8 mSv as opposed to 1.6 mSv (*P* < 0.001). This underscores the importance of adhering to the appropriate scanning protocols to minimize radiation exposure in pediatric patients.^31^

Notably, some technologists applied identical parameters to both adult and pediatric patients, further underscoring the need for specialized training among technologists to mitigate radiation exposure risks. Notably, studies conducted by ref. 7 Indicated that the hospitals under examination did not employ shielding measures such as thyroid, gonad, or eye lens shields to safeguard radiosensitive organs from scattered radiation. This highlights an area where improvements in radiation safety practices are required to enhance patient protection. Previous research has revealed significant disparities in screening duration, number of radiographic images taken, dose-length product, and effective dose in pediatric CT scans. These discrepancies highlight the trend of excessive and unwarranted radiation exposure in pediatric patients, indicating a lack of optimization in current clinical practices. Notably, the study also identified relatively minor variations in radiation doses for brain and chest scans but significant discrepancies in abdominal scans. These variations may be attributed to diverse clinical indications. Nevertheless, a crucial challenge is the insufficient knowledge of technologists, radiologists, and physicians regarding the capabilities of CT machines and their associated radiation risks. Despite the undeniable benefits of CT scans in pediatric medicine, there is an urgent and compelling need for enhanced training and optimization measures to minimize avoidable radiation exposure in pediatric patients.^7^ It has been emphasized that patient motion could contribute to the need for rescanning. This observation was subsequently reinforced by ref. 38, who determined that CT scans were repeated primarily in cases involving older patients, females, and individuals with higher body mass indices due to patient movement. These studies collectively underscore the various reasons behind the repetition of CT scans, highlighting unnecessary radiation exposure, which can compromise patient satisfaction and health as well as waste valuable time and resources. Therefore, it is imperative to recognize the distinctive nature of CT scanning and consider these factors when developing strategies to minimize the repetition of scans, ultimately prioritizing patient well-being and radiation safety.

## Discussion

This systematic review was undertaken to synthesize the existing evidence on strategies to minimize radiation exposure risks from CT scans in pediatric patients. A comprehensive literature search identified 30 studies that met the eligibility criteria, providing insights into the cancer risks of CT radiation and approaches to reduce unnecessary exposure in pediatric patients. However, the included studies demonstrated heterogeneity in study populations, interventions, and measured outcomes. Additionally, there is a lack of randomized controlled trials that specifically examine radiation-reduction strategies.

Growing concern regarding the potential cancer risks associated with repeated CT scan exposure in pediatric patients has increased substantial research interest. Accumulating evidence strongly suggests that an increased frequency of CT scans in pediatric patients leads to a higher cumulative radiation exposure, which is closely linked to an elevated average cancer risk.^7^ Consequently, owing to the heightened incidence of cancer associated with this exposure, CT scans have been associated with a significant increase in the number of cancer cases.

Our meta-analysis demonstrated a pooled RR of 1.49 (95% CI: 1.44-1.54) for overall cancer risk associated with CT exposure, consistent with previous evidence. For example, Huang et al,^39^ reported a comparable 1.32-fold increased cancer risk among more than 1.1 million CT-exposed children, reinforcing confidence in the association. For specific cancer types, we observed a significantly elevated risk of brain tumors (RR = 1.55, 95% CI: 1.22-1.97) but a non-significant association with leukemia (RR = 1.23, 95% CI: 0.72-2.12). This pattern aligns with Pearce et al.’s landmark UK cohort study, which reported excess relative risks of 0.023 per mGy for brain tumors and 0.036 per mGy for leukemia.^9^ However, more recent evidence from the 2023 EPI-CT European cohort found an excess relative risk of 1.96 per 100 mGy for hematological malignancies, indicating that leukemia risk may be greater than earlier estimates with extended follow-up.^40^ Differences between our leukemia findings and previous studies may reflect variations in sample size, duration of follow-up, and radiation dose assessment methods.

The dose-response relationship we observed, with multiple CT scans (RR = 2.51 for ≥2 scans) significantly increasing cancer risk compared with a single scan (RR = 1.07), is consistent with findings from previous studies. Huang et al,^6^ and Mathews et al,^5^ similarly reported cumulative dose effects, noting that brain tumor risk can increase two-to four-fold at cumulative doses above 120 mGy.^15^ This collective evidence strongly reinforces the ALARA principle and highlights the importance of avoiding unnecessary repeat scans, particularly in younger children who have the greatest lifetime risk. Clinically, these findings underscore the need to apply the ALARA (as low as reasonably achievable) principle when using CT imaging in pediatrics. Dose-optimization strategies should be rigorously implemented,^5^ including the use of child-specific protocols, adjustment of scanner settings based on patient size, minimizing scan range, and shielding radiosensitive tissues.^15^

Among the most impactful technological advances in pediatric CT dose reduction is iterative reconstruction (IR), which has transformed how radiation exposure is managed. Compared with traditional filtered back projection (FBP), IR algorithms can reduce radiation dose by 30-50% while preserving diagnostic image quality.^41^ More advanced approaches, such as model-based iterative reconstruction (MBIR) and deep learning image reconstruction (DLR) have demonstrated even greater dose savings, achieving reductions of 50-80% relative to standard protocols.^42,43^ A 2023 systematic review further showed that DLR outperforms conventional IR in pediatric head and cardiac CT, offering superior image quality at substantially lower doses.^44^ Despite these benefits, clinicians should be aware that IR techniques can introduce a “smoothed” appearance and may reduce low-contrast spatial resolution, highlighting the need for careful protocol optimization.^42^ Additional dose-reduction innovations, including automatic exposure control systems, low-tube-voltage techniques (eg, 80 kVp), and emerging photon-counting detector CT also show considerable promise for enhancing safety in pediatric imaging.^45^ Overall, the implementation of these dose-optimization strategies, particularly IR and DLR methods, should be prioritized across pediatric CT services to minimize radiation exposure and reduce long-term cancer risk from medically necessary scans.

Our review identified protocol variability, insufficient staff training, and motion artifacts as major contributors to unnecessary repeat CT scans. These findings are consistent with previous literature but must be interpreted within the context of current evidence-based strategies aimed at reducing scan repetition. To address motion artifacts, one of the most common causes of rescanning, multiple non-sedation strategies have been developed. A recent 2025 quality improvement study demonstrated that adopting volumetric target-mode ECG-gated CT protocols significantly reduced scan times in children aged 0-4 years, thereby decreasing motion artifacts without increasing the need for sedation.^46^ Advances in modern multislice CT systems, including sub-second gantry rotation times, now allow many pediatric examinations to be completed within a single breath-hold or with only minimal sedation, reducing reliance on deeper anesthesia.^47^ Non-pharmacological techniques, such as “feed and swaddle” approaches for infants, behavioral rehearsal programs for toddlers, and child life specialist support, have also been shown to reduce motion-related scan repetition.^48^ When sedation is not desirable, pediatric-specific immobilization devices, such as vacuum bean bags and Velcro-based restraints—offer additional means of minimizing patient movement.^49^

Protocol standardization remains an unmet need. Evidence shows that implementing pediatric-specific CT protocols can reduce radiation doses by up to 50% while preserving diagnostic quality.^7^ Despite this, our review indicates that many institutions, especially non-specialized centers continue to use adult protocols for pediatric patients. Integration of decision support systems into CT ordering workflows has reduced inappropriate imaging by 20-30% in several settings^50^ suggesting that electronic clinical decision support may help prevent not only unnecessary initial scans but also avoidable repeat examinations. Future research should prioritize development of automated image-quality prediction tools capable of assessing scan adequacy in real time, which could reduce rescanning. Additionally, establishing international consensus guidelines for pediatric CT protocol standardization is essential to ensure consistent, safe, and high-quality imaging across diverse clinical environments.

Dose-optimization interventions specifically aimed at minimizing repetitive CT scans in pediatric patients require further study. Based on this evidence, potential strategies include checklists to justify CT use, the use of MRI as an alternative when feasible, defined criteria for scan repetition, mandatory second-reader confirmation before repeats, and peer review of protocols generating excessive repeats. Studying such targeted interventions can strengthen the evidence for minimizing repetitive CT scans and radiation exposure in pediatric patients.

This systematic review and meta-analysis had several limitations. First, there was a high heterogeneity in the selection of variables used to assess cancer risk across the included studies. This variability makes it challenging to establish consistent and comparable measures of cancer risk, compromising the reliability and generalizability of the findings. Therefore, caution should be exercised when concluding this study. Moreover, a notable limitation arises from the use of simulation or mathematical phantoms in some of the included studies to estimate organ doses rather than directly measuring them in pediatric patients. Although these methods are commonly used for radiation dose estimation, they introduce inherent uncertainty. Simulated organ doses may not fully capture individual variations in patient body size, anatomy, or other factors that can influence the actual organ doses. This reliance on simulated organ doses presents a substantial obstacle in accurately assessing the relationship between CT scan repetition and cancer risk in pediatric patients within the context of this review. In addition to these limitations, another significant constraint was the lack of clear explanations for why CT scans were repeated in some of the included studies. Understanding the underlying reasons for, and justifications for, repeated scans is pivotal for evaluating the potential impact of cumulative radiation exposure on cancer risk. Unfortunately, most studies have not offered explicit insights into the motivations for repeated CT scans. This lack of clarity hampers our ability to draw definitive conclusions regarding the association between CT scan repetition, cumulative dose, and its potential implications for cancer risk in pediatric patients. To address these limitations and advance our understanding of this critical area, future research should prioritize comprehensive reporting and in-depth investigations. A thorough exploration of the motivations and clinical justifications behind repeated CT scans in pediatric patients is essential to accurately determine the complex relationship between CT scan repetition and cancer risk.

## Conclusion

This systematic review and meta-analysis provide compelling evidence that pediatric CT radiation exposure is associated with a significantly increased risk of cancer. Children exposed to CT scans had a 1.49-fold higher overall cancer risk compared with unexposed children (95% CI: 1.44-1.54). A clear dose-response pattern emerged, with children undergoing two or more CT scans experiencing more than double the cancer risk (RR = 2.51) relative to those with a single scan. Elevated risk was particularly evident for brain tumors (RR = 1.55), whereas evidence for leukemia was less consistent, indicating the need for careful evaluation of head CT use.

These findings highlight the need for stricter justification before ordering CT scans in children, especially repeat imaging, along with implementing standardized pediatric protocols that incorporate dose-reduction technologies. Greater use of non-ionizing alternatives when clinically appropriate and improved training for radiology staff are also essential. Future research should prioritize developing robust, evidence-based guidance on the appropriateness of repeat CT imaging and optimizing age-specific radiation dose strategies to ensure diagnostic value while minimizing long-term cancer risk.

## Supplemental Material

**Supplemental Material -** Factors Associated With CT Scan Repetition in Pediatrics and Its Relationship With Cancer Risk: A Systematic Review and Meta-Analysis Supplemental Material for Factors Associated With CT Scan Repetition in Pediatrics and Its Relationship With Cancer Risk: A Systematic Review and Meta-Analysis by Tahani Al-Shangeeti, Rozilawati Ahmad, Salah A. Alshehade, Maisa Elzaki, Fahad A. Alatheem, Wala Al-Sharif, Khalid Suliman, Amjad Al-Shangeeti, Bandar Al-Shamrani, Abdullah Hanbali, Tariq Almuzaini, Mohammed Alnasser, Taher Althubyani, Abdullah Aljohani, Ahmed Mahdi Baba, Amal Zaki Alruwaili, Hamid Osman Hamid Osman and Mohammed Abdullah Alshawsh in Dose-Response.

## Data Availability

All data analyzed in this systematic review and meta-analysis are from published studies cited in the references. The datasets used for the meta-analysis are available from the corresponding author upon reasonable request.*

## Appendix

### List of Abbreviations

ALARA: As low as reasonably achievable
CI: Confidence interval
CNS: Central nervous system
CT: Computed tomography
ERR: Excess relative risk
GCS: Glasgow coma scale
HR: Hazard ratio
LAR: Lifetime attributable risk
MDS: Myelodysplastic syndromes
mGy: Milligray
mSv: Millisievert
NOS: Newcastle-Ottawa scale
OR: Odds ratio
PET: Positron emission tomography
PRISMA: Preferred reporting items for systematic reviews and meta-analyses
PROSPERO: International prospective register of systematic reviews
RR: Risk ratio
SFDA: Saudi food and drug authority
SPECT: Single-photon emission computed tomography
UK: United Kingdom
USA: United States of America
