# Supplementary material for: Factors Associated With CT Scan Repetition in Pediatrics and Its Relationship With Cancer Risk: A Systematic Review and Meta-Analysis
Source: Dose Response. 2026 Apr 16;24(2):15593258261419666. doi: 10.1177/15593258261419666 (PMC13100438; doi:10.1177/15593258261419666)
Supplement: Supplemental Material - Factors Associated With CT Scan Repetition in Pediatrics and Its Relationship With Cancer Risk: A Systematic Review and Meta-Analysis [file sj-pdf-1-dos-10.1177_15593258261419666.pdf]

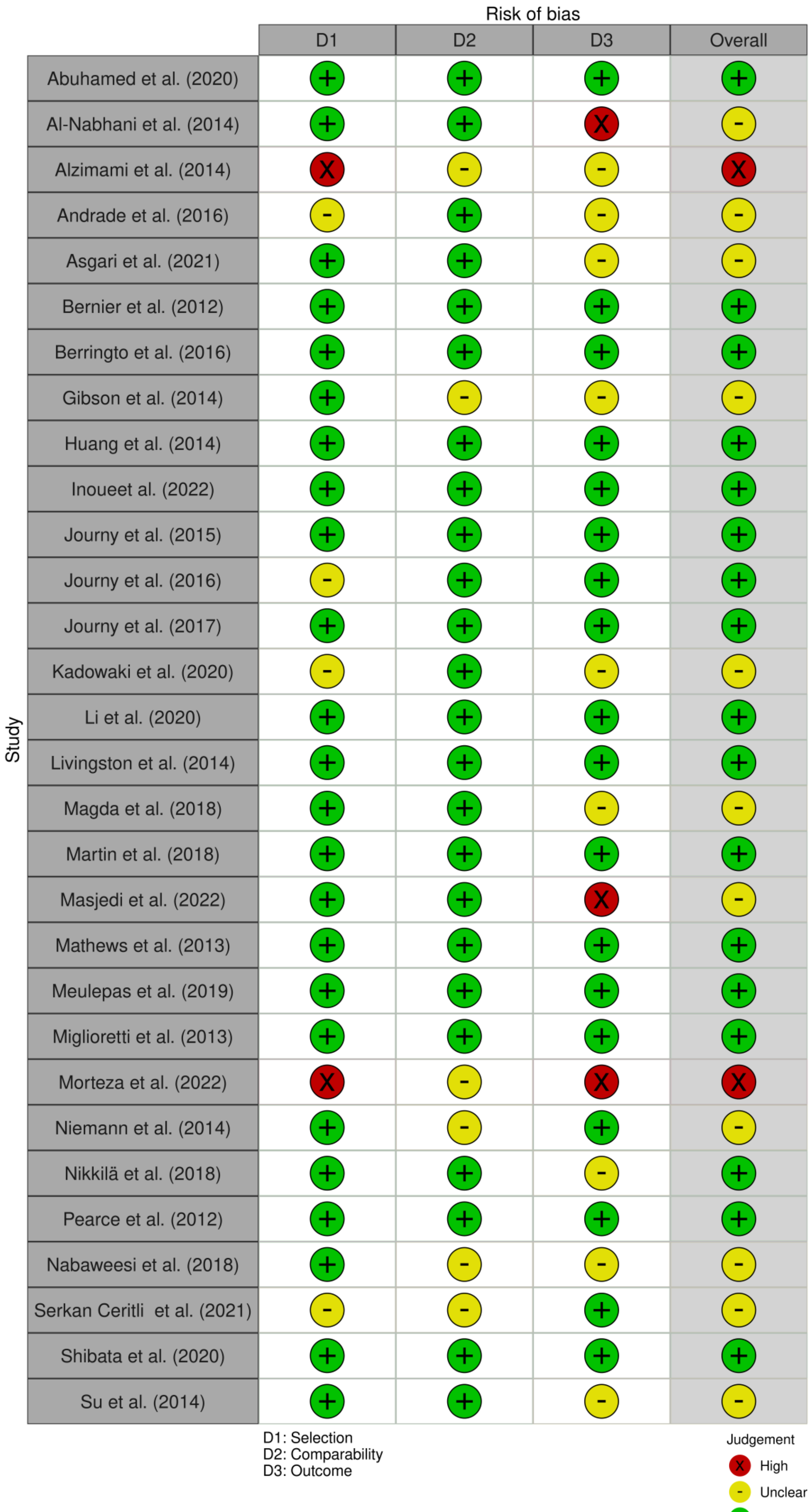

Risk of bias
D1 D2 D3 Overall
Study
Abuhamed et al. (2020) + + + +
Al-Nabhani et al. (2014) + + X -
Alzimami et al. (2014) X - - X
Andrade et al. (2016) - + - -
Asgari et al. (2021) + + - -
Bernier et al. (2012) + + + +
Berringto et al. (2016) + + + +
Gibson et al. (2014) + - - -
Huang et al. (2014) + + + +
Inoueet al. (2022) + + + +
Journy et al. (2015) + + + +
Journy et al. (2016) - + + +
Journy et al. (2017) + + + +
Kadowaki et al. (2020) - + - -
Li et al. (2020) + + + +
Livingston et al. (2014) + + + +
Magda et al. (2018) + + - -
Martin et al. (2018) + + + +
Masjedi et al. (2022) + + X -
Mathews et al. (2013) + + + +
Meulepas et al. (2019) + + + +
Miglioretti et al. (2013) + + + +
Morteza et al. (2022) X - X X
Niemann et al. (2014) + - + -
Nikkilä et al. (2018) + + - +
Pearce et al. (2012) + + + +
Nabaweesi et al. (2018) + - - -
Serkan Ceritli et al. (2021) - - + -
Shibata et al. (2020) + + + +
Su et al. (2014) + + - -
D1: Selection
D2: Comparability
D3: Outcome
Judgement
X High
- Unclear
